# Supplementary material for: Acetylation of KLF5 maintains EMT and tumorigenicity to cause chemoresistant bone metastasis in prostate cancer
Source: Nat Commun. 2021 Mar 17;12:1714. doi: 10.1038/s41467-021-21976-w (PMC7969754; doi:10.1038/s41467-021-21976-w)
Supplement: Supplementary file 1 — Supplementary Information [file 41467_2021_21976_MOESM1_ESM.pdf]

Supplementary information

Acetylation of KLF5 causes chemoresistant bone metastasis by activating CXCR4/IL-11 while maintaining EMT and tumorigenicity in prostate cancer

Zhang et al.

I. **Supplementary figures**
II. **Supplementary tables**

I. Supplementary figures

Supplementary figure 1

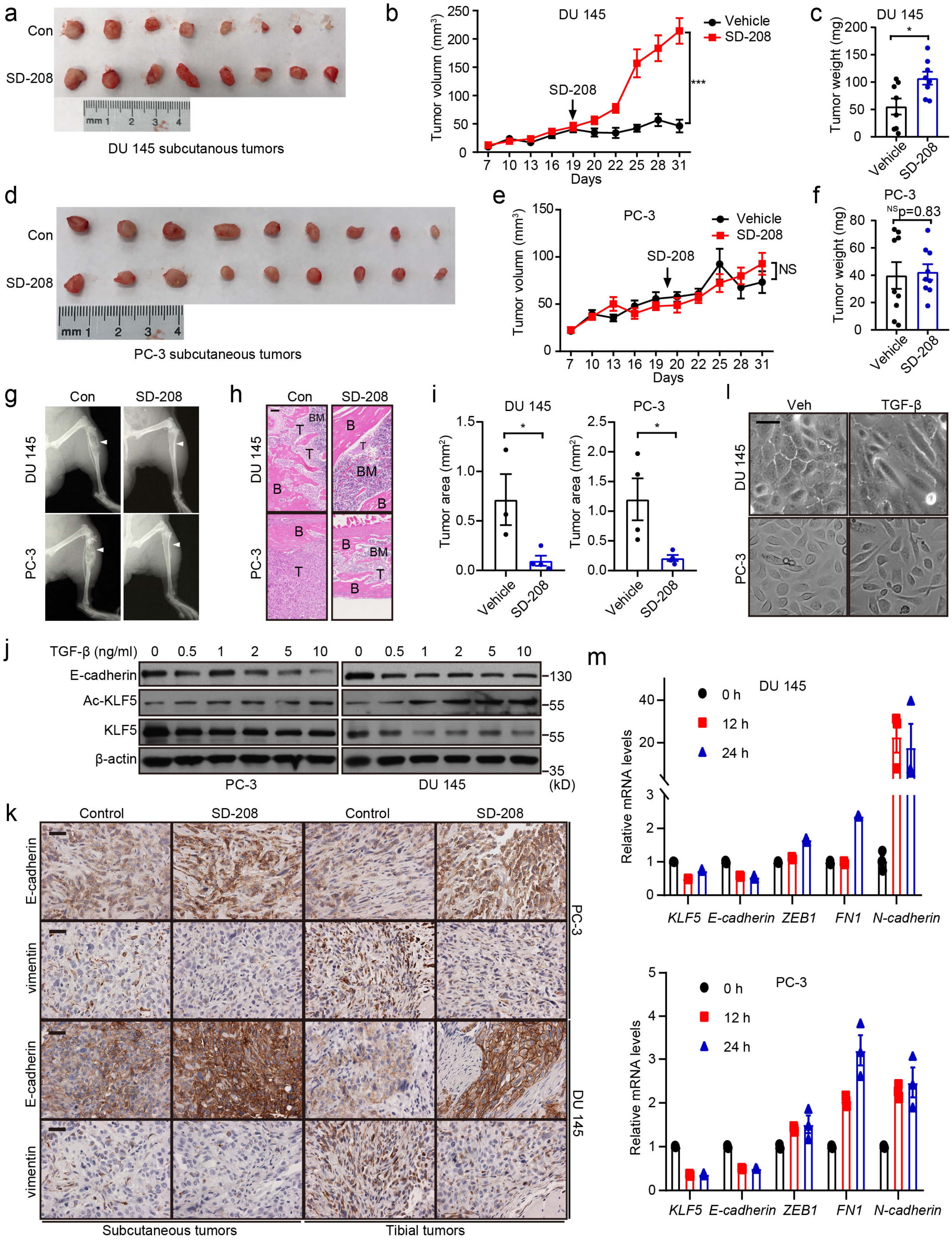

Supplementary figure 1. Inhibition of TGF-β signaling by SD-208 promotes subcutaneous tumor growth but suppresses tibial tumor growth and restores epithelial phenotypes. (a-f) Subcutaneous tumor growth of DU 145 (a-c) and PC-3 (d-f) cells with or without SD-208 treatment (50 mg/kg/day), as indicated by tumor images (a, d), volume-based growth curves (b, e), and tumor weights at excision (c, f). n=8 tumors per group for DU 145 (b, c) while n=9 tumors per group for PC-3 (e, f). (g-i) SD-208 inhibits tibial tumor growth in both DU 145 and PC-3 cells, as indicated by X-ray radiographs (g), H&E staining (h), and quantification of tumor areas in tibia (i). For DU 145 tibial tumors, n=3 tumors for vehicle group and n=4 tumors for SD-208 group. For PC-3 tibial tumors, n=4 tumors for each group. White arrows indicate bone lesions. B, trabecular bone region; BM, bone marrow region; T, tumor region. (j) Detection of indicated proteins by Western blotting in whole cell lysates of TGF-β treated PC-3 and DU 145 cells for 24 hours *in vitro*. (k) IHC staining of EMT markers (epithelial marker E-cadherin and mesenchymal marker vimentin) in subcutaneous and tibial tumors of PC-3 and DU 145 cells from mice treated with the SD-208 TGF-β inhibitor. Scale bars, 50 µm. (l, m) TGF-β induces EMT in PC-3 and DU 145 cells *in vitro*, as indicated by the spindle-like morphology (l) and expression changes in EMT markers detected by real-time qPCR (m). *E-cadherin* is an epithelial marker, while *ZEB1*, *FN1* and *N-cadherin* are mesenchymal markers. n=3 technical replicates. In panels **c**, **f** and **i**, data are shown in mean ± S.E.M. *, $p < 0.05$; ***, $p<0.001$; NS, not significant. Two-way ANOVA assay were performed in **b** and **e**. Two-tailed Student's t-test were used for other statistical analyses. The experiments in panels **j**, **k** and **l** were repeated at least twice and consistent results were achieved. Source data are provided as a Source Data file.

Supplementary figure 2

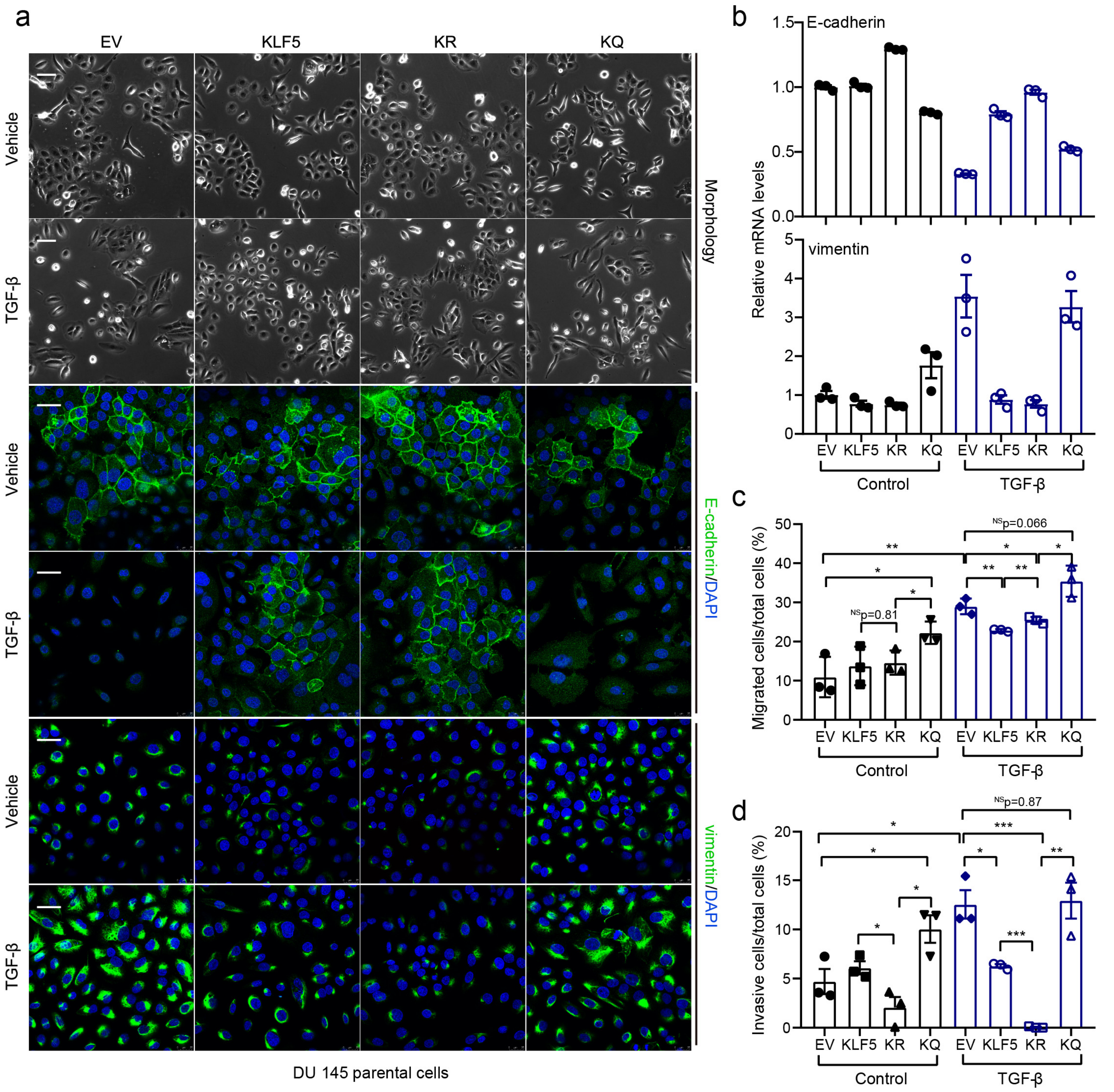

Supplementary figure 2. KLF5 acetylation at K369 is essential for TGF-β to induce EMT in DU 145 parental cells. (a, b) Detection of cell morphology using phase contrast microscopy and EMT markers using immunofluorescent (IF) staining (green) (a) and real-time qPCR (b) in TGF-β-treated or not treated DU 145 parental cells in which wildtype *KLF5* (KLF5), acetylation-deficient mutant $KLF5^{KR}$ ($\mathrm{KLF5^{KR}}$ or KR), Ac-KLF5-mimicking mutant $KLF5^{KQ}$ ($\mathrm{KLF5^{KQ}}$ or KQ), or empty vector (EV) were ectopically expressed. Scale bars, 50 µm. E-cadherin is an epithelial marker and vimentin is a mesenchymal marker. (c, d) Analyses of migration (c) and invasion (d) in the same cells from panel **a** using the Boyden chamber assay. n=3 chambers for each condition. In panels **c** and **d**, data are shown in mean ± S.E.M. *, p < 0.05; **, p < 0.01; ***, p<0.001; NS, not significant (two-tailed Student's t-test). Source data are provided as a Source Data file.

Supplementary figure 3

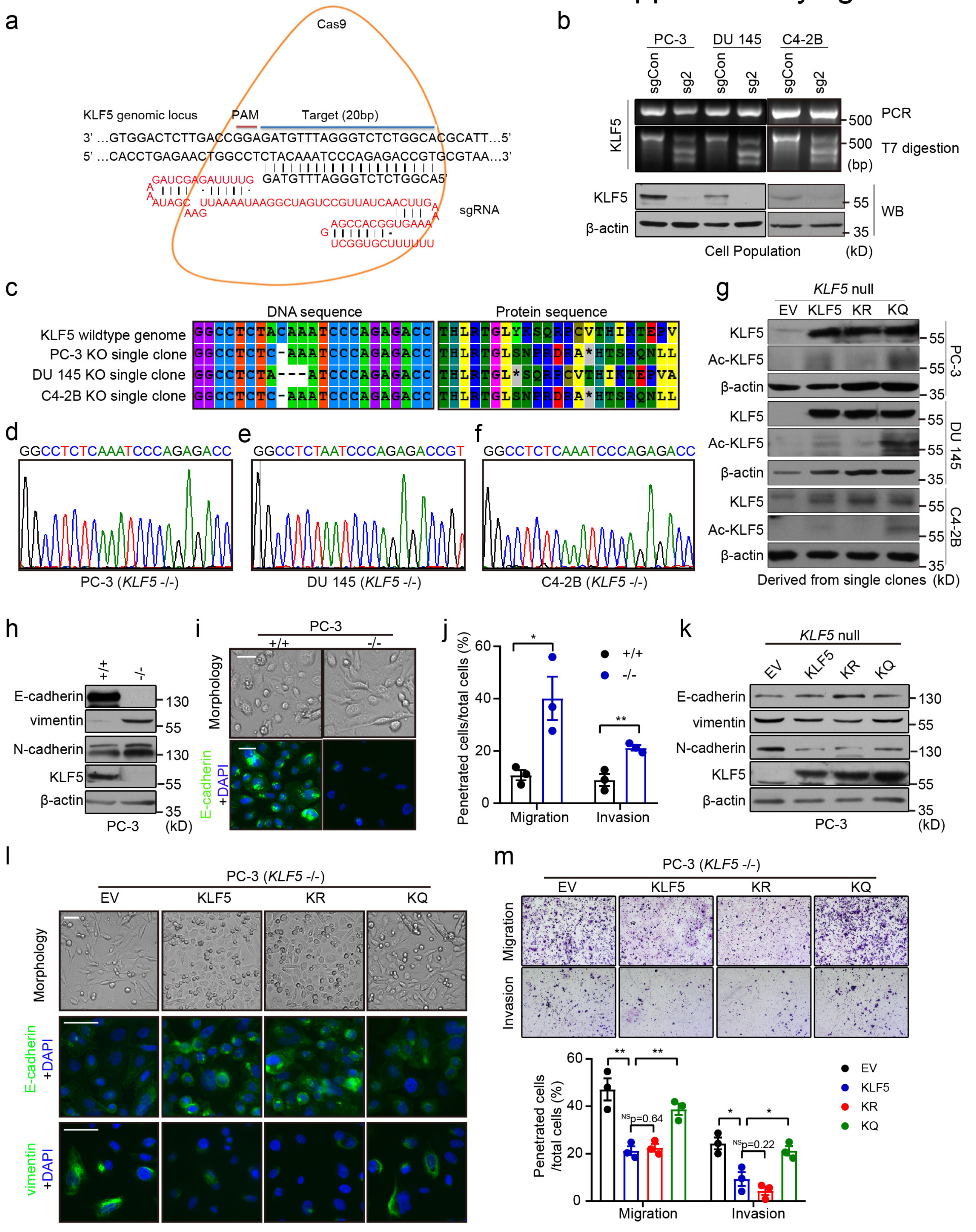

Supplementary figure 3. Establishment and EMT features of prostate cancer cells with different KLF5 statuses. (a-f) Establishment of PC-3, DU 145 and C4-2B cell lines in which endogenous *KLF5* was deleted using a CRISPR-cas9 system. In the design of targeting vector (a), the single guide RNA (sgRNA) is marked in red. Successful targeting of *KLF5* was confirmed by T7 digestion and the absence of KLF5 expression by Western blotting (WB) (b), and by sequencing PCR products for cell clones (c-f). A clone of PC-3 cells and a clone of C4-2B cells with a deletion of one nucleotide (i.e., A) respectively and a clone of DU 145 cells with a deletion of 3 nucleotides (i.e., ACA) were used for subsequent experiments, and both deletions occurred in both KLF5 alleles to cause either a frame shift (i.e., PC-3 and C4-2B) or early stop codon (i.e., DU 145) in KLF5 protein translation. *, stop codon. (g) Wildtype KLF5, $KLF5^{K369R}$ (KR), $KLF5^{K369Q}$ (KQ), and empty vector (EV) were ectopically expressed by lentiviral infection, as confirmed by WB with the KLF5 and Ac-KLF5 antibodies. (h-j) Deletion of *KLF5* induced EMT in PC-3 cells, as indicated by marker expression (Western blotting) (h), morphological changes (i), IF staining of E-cadherin (i), and migration and invasion analyses (j). +/+, KLF5 wildtype; -/-, KLF5 knockout. (k-m) $KLF5^{KR}$ prevented EMT, as indicated by expression of EMT markers (Western blotting) (k), morphology and IF staining of EMT markers (l), and cell motility and invasion (m). Epithelial marker was E-cadherin, while the mesenchymal markers were vimentin and N-cadherin. Cell motility and invasion were determined by the Boyden chamber transwell assay. n=3 chambers for each condition. In panels **j** and **m**, data are shown in mean ± S.E.M. Scale bars, 50 µm. *, $p < 0.05$; **, $p < 0.01$ (two-tailed Student's t-test). Source data are provided as a Source Data file.

Supplementary figure 4

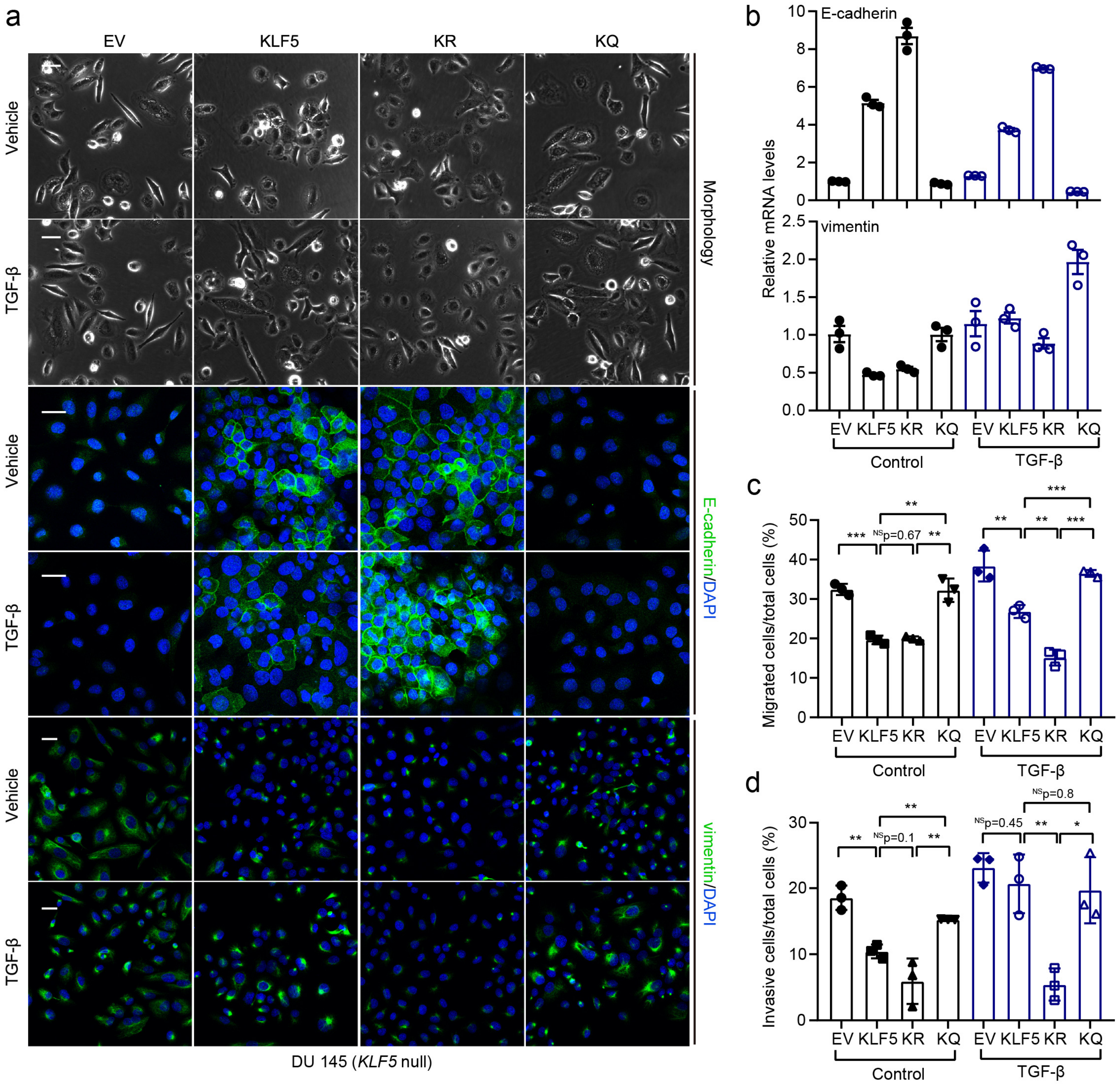

Supplementary figure 4. Loss of KLF5 induces EMT and deacetylated KLF5 restores epithelial features. (a, b) Detection of cell morphology using phase contrast microscopy and EMT markers using immunofluorescent (IF) staining (green) (a) and real-time qPCR (b) in TGF-β-treated or not treated KLF5-null DU 145 cells in which wildtype *KLF5* (KLF5), acetylation-deficient mutant $KLF5^{KR}$ ($KLF5^{KR}$ or KR), Ac-KLF5-mimicking mutant $KLF5^{KQ}$ ($KLF5^{KQ}$ or KQ), or empty vector (EV) were ectopically expressed. Scale bars, 50 μm. E-cadherin is an epithelial marker and vimentin is a mesenchymal marker. (c, d) Analyses of migration (c) and invasion (d) in the same cells from panel **a** using the Boyden chamber assay. n=3 chambers for each condition. In panels c and d, data are shown in mean ± S.E.M. *, $p < 0.05$; **, $p < 0.01$; ***, $p<0.001$; NS, not significant (two-tailed Student's t-test). Source data are provided as a Source Data file.

Supplementary figure 5

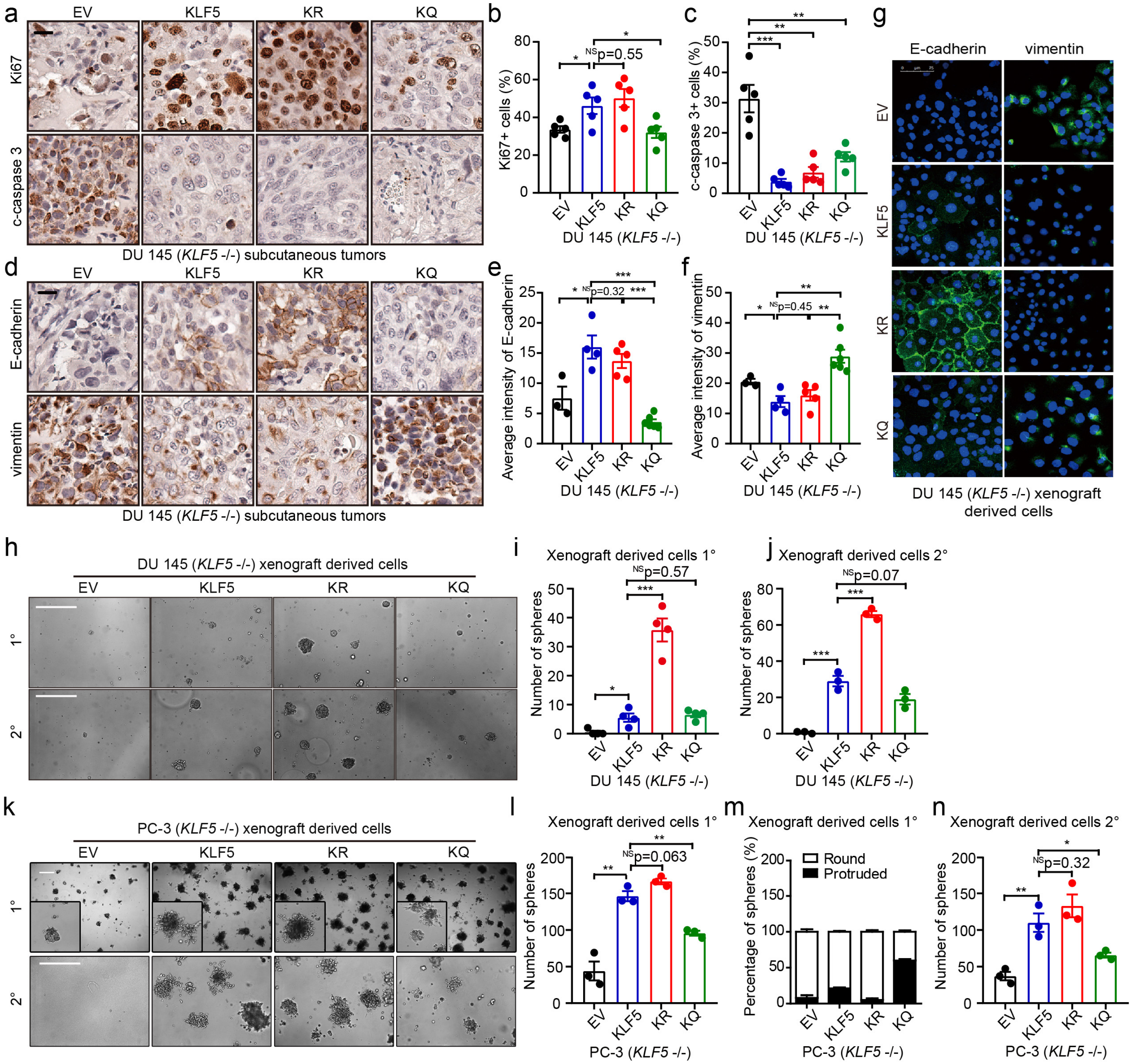

Supplementary figure 5. Acetylation of KLF5 maintains the mesenchymal phenotype while slowing sphere formation of PCa cells. (a-c) IHC staining of proliferation marker Ki67 and apoptosis marker cleaved-caspase 3 (c-caspase 3) in subcutaneous tumors of DU 145 (a), and statistical analysis of Ki67 (b) and c-caspase-3 (c) positive rates in the tumors. n=5 tumors for each group. (d-f) IHC staining (d) and quantitative analyses of average intensities for E-cadherin (e) and vimentin (f) by Fiji software in DU 145 subcutaneous tumors with different forms of KLF5. n=3, 4, 5, 6 tumors for EV, KLF5, KR and KQ group respectively. Scale bars in **a** and **d**, 50 µm. (g) Detection of EMT marker expression by IF staining in primary cultures established from DU 145 subcutaneous tumors. Scale bar, 75 µm. (h-n) Sphere formation capability in Matrigel for cells established from DU 145 (h-j) and PC-3 (k-n) subcutaneous tumors, as indicated by representative sphere images (h, k) and sphere numbers (i, j, l and n). Protruded spheres (m) are those with branches in their peripheral zones. 1°, the first round/generation of culture; 2°, the second round/generation of culture. Scale bars, 500 µm. n=4 wells in **i**; n=3 wells in **j** and **l**; n=3 wells in **n**. In panels **b**-**c**, **e**-**f**, **i**-**j**, **l**-**n**, data are shown in mean ± S.E.M. *, $p < 0.05$; **, $p < 0.01$; ***, $p<0.001$; NS, not significant (two-tailed Student's t-test). Source data are provided as a Source Data file.

Supplementary figure 6

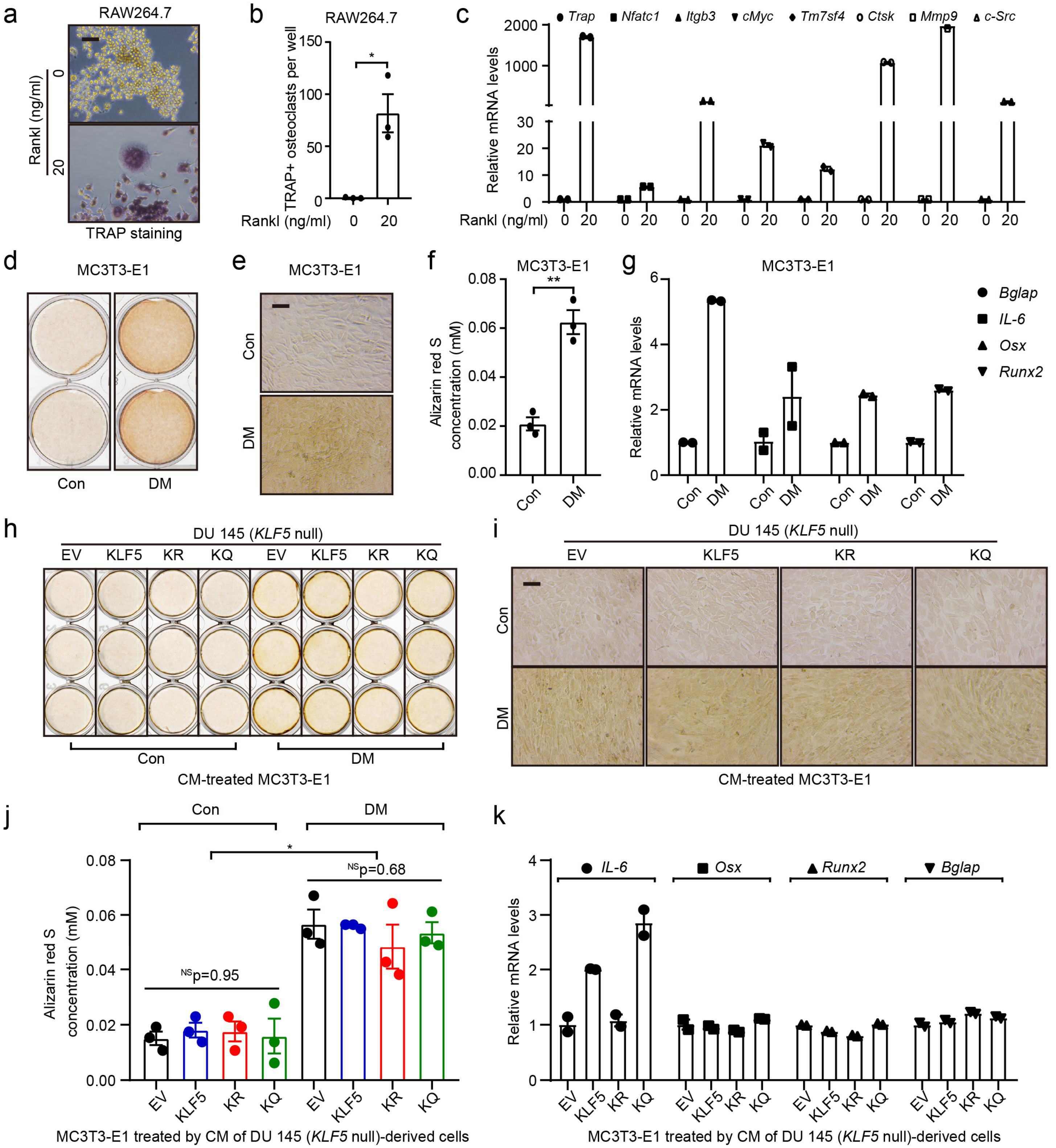

Supplementary figure 6. Acetylation of KLF5 in tumor cells induces IL-6 expression in osteoblasts in co-culture. (a-c) RAW264.7 pre-osteoclasts were treated with Rankl at 20 ng/ml for 6 days, and osteoclast differentiation was indicated by TRAP staining (a), statistical analysis of TRAP+ osteoclasts (b), and expression of osteoclast differentiation markers detected by real-time qPCR (c). TRAP+ osteoclasts were analyzed in 3 different wells in **b**. (d-g) Mouse MC3T3-E1 osteoblasts were treated with differentiation medium (DM, containing 50 μg/ml ascorbic acid and 10 mM β-

glycerophosphate) for 16 days, which were then subjected to the Alizarin Red S assay to determination the calcium content (d-f) and real-time qPCR to detect the expression of osteoblast differentiation markers *Bglap*, *IL6*, *Osx* and *Runx2* (g). Con, control. Panels **d** and **e** are photos of Alizarin Red S stained cells at different magnifications. Alizarin Red S concentrations were measured in 3 different wells in **f**. (h-k) MC3T3-E1 cells were treated with CM from DU 145 cells with different acetylation statuses of KLF5 in the context of DM or control medium (Con); and to test their osteoblast differentiation, these cells were then subjected to the Alizarin Red S assay to determine the calcium content (h-j) and real-time qPCR to detect the expression of osteoblast differentiation markers (k). Panels **h** and **i** are photos of Alizarin Red S stained cells at different magnifications. Alizarin Red S concentrations were measured in 3 different wells in **j**. Scale bars, 50 µm. In panels **b**, **f** and **j**, data are shown in mean ± S.E.M. *, $p < 0.05$; **, $p < 0.01$; NS, not significant (two-tailed Student's t-test). Representative images are shown in **a**, **e** and **i**, and statistically analyzed in **b**, **f** and **j**, respectively. Source data are provided as a Source Data file.

Supplementary figure 7

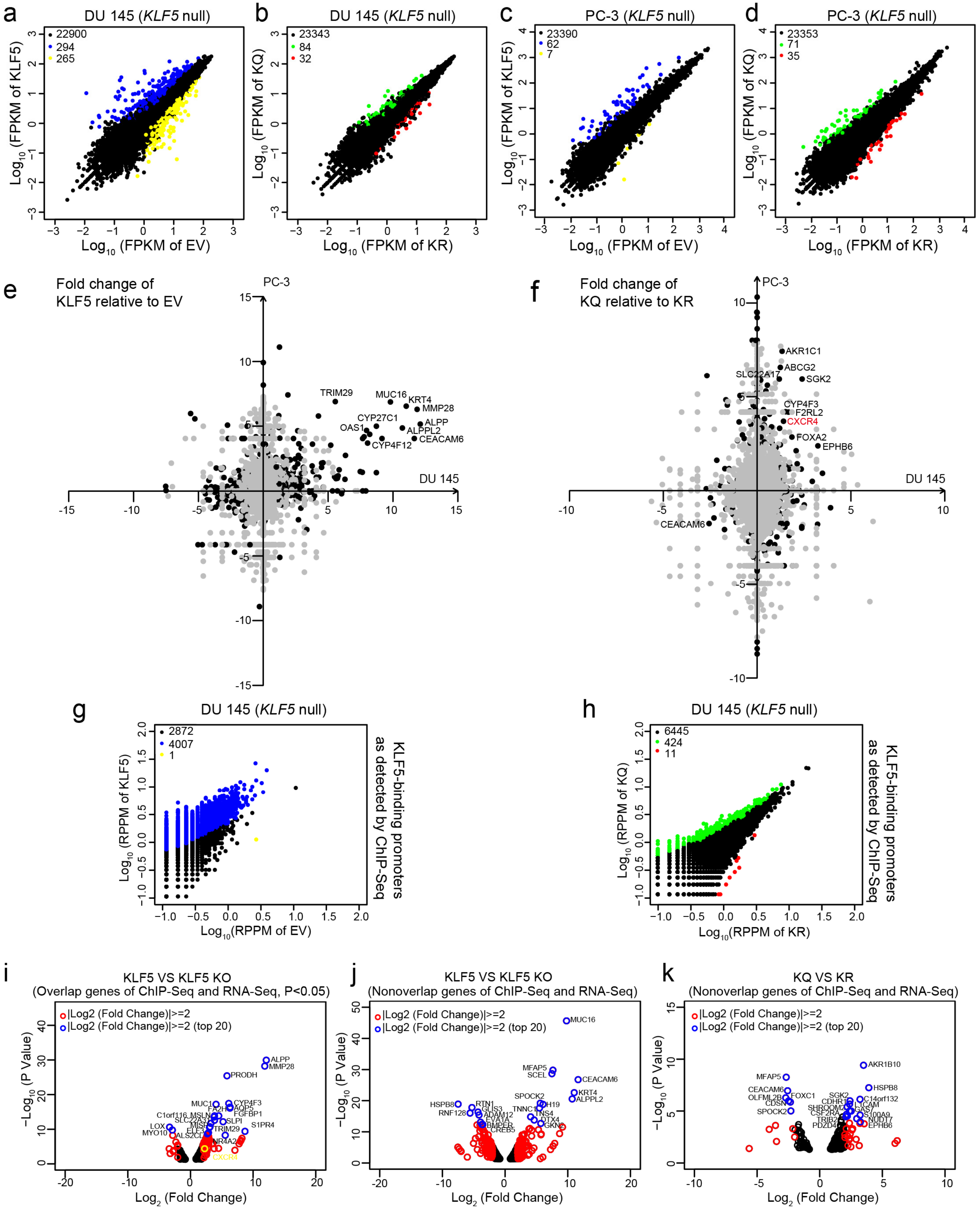

Supplementary figure 7. KLF5^{KQ} (KQ) and KLF5^{KR} (KR) regulate distinct sets of genes in PCa cells. RNA-Seq and ChIP-Seq analyses were performed using KLF5-null DU 145 and PC-3 cells that ectopically expressed empty vector (EV), wildtype *KLF5* (KLF5); *KLF5*^{K369R} (KR), and *KLF5*^{K369Q} (KQ). (a-d) Differentially expressed genes between EV and KLF5 (a, c) or between KR and KQ (b, d) in DU 145 (a, b) or PC-3 (c, d) cells. Details are available in Supplementary dataset 1 for DU 145 and in Supplementary dataset 2 for PC-3 cells. Blue and yellow dots in **a** and **c** indicate the genes that were upregulated and downregulated, respectively, by KLF5 by at least 2-fold; while green and red dots in **b** and **d** indicate genes that were upregulated by KQ and KR, respectively, by at least 2-fold. The FDR adjusted p-value for the changes between EV and KLF5 or between KR and KQ was no greater than 0.001. Black dots indicate other genes in the UCSC HG19 entries. FPKM, fragments per kilobase of exon per million mapped reads. (e, f) Overlap of differentially expressed genes between EV and KLF5 (e, details are available in Supplementary dataset 3) and between KR and KQ (f, details are available in Supplementary dataset 4). Black dots here indicate differentially expressed genes with FDR adjusted p-value less than 0.001 in either DU 145 or PC-3 cells, and grey dots indicate other genes in the UCSC HG19 entries. The top ten differentially expressed genes with the same trends between DU 145 and PC-3 cell lines are shown in panels **e** and **f**. (g, h) Promoter regions (-2500~+500) bound by KLF5 (g) and differentially bound promoter regions between KR and KQ (h), as detected by ChIP-Seq analysis using the KLF5 antibody. Blue dots and yellow dots in **g** indicate the enriched peaks in KLF5 and EV group, respectively, while green and red dots in **h** indicate KQ- and KR-enriched binding peaks, respectively. All dots with the 4 colors indicate binding peaks with p-values not greater than 0.01 and fold changes not less than 1.5. Black dots indicate other peaks that occurred in the promoter regions in the ChIP-Seq analysis. RPPM, reads per peak per million. Details are available in Supplementary dataset 5. (i) Overlapping genes between RNA-Seq and ChIP-Seq analyses in the EV and KLF5 comparison of DU 145 cells. Circles indicate the genes that had both binding peaks in their promoter regions and expression changes with a p-value no more than 0.05. Details are available in Supplementary dataset 6. (j, k) Non-overlapping genes between RNA-Seq versus ChIP-Seq for the EV and KLF5 comparison (j, details are available in Supplementary dataset 8) and the KR and KQ comparison (k, details are available in Supplementary dataset 9) of DU 145 cells. Circles indicate genes that did not have binding peaks in the ChIP-Seq analysis but had expression changes with p-values no more than 0.05. In **i**-**k**, blue and red dots indicate genes with expression changes no less than 4-fold, and blue dots indicate the 20 genes with the greatest fold changes.

Supplementary figure 8

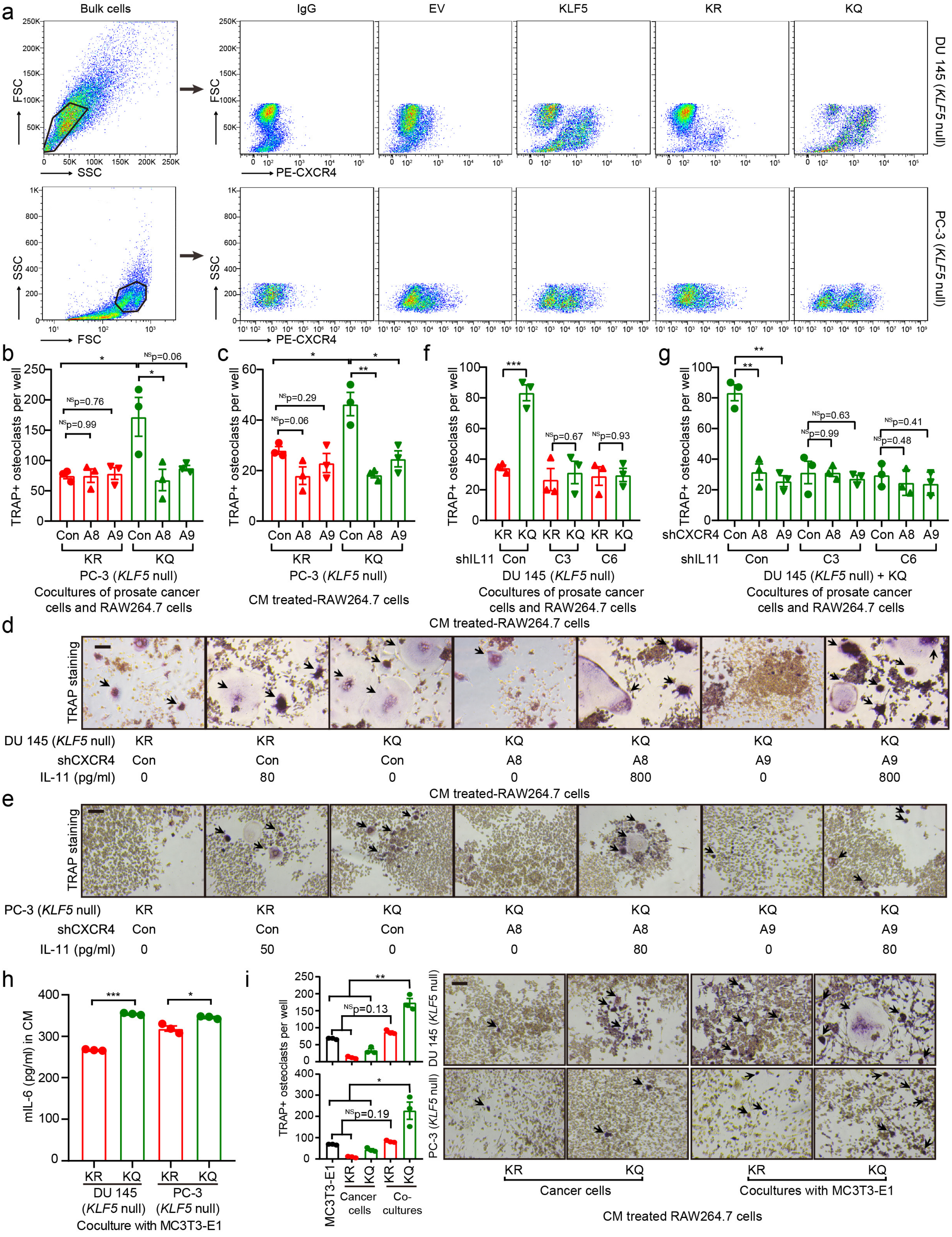

Supplementary figure 8. IL-11 is required for the Ac-KLF5/CXCR4 axis in PCa cells to promote osteoclast differentiation. (a) Gating strategies and pseudocolor plots for the analysis in **Figures 6c, d**. (b, c) Knockdown of *CXCR4* suppressed the differentiation of RAW264.7 pre-osteoclasts induced by their co-culture with (b) or by CM from (c) KQ-expressing PC-3 cells, as indicated by TRAP staining. n=3 wells per group. (d, e) Addition of IL-11 rescued the decrease in osteoclast differentiation of RAW264.7 cells by the CM from DU 145 cells (d) and PC-3 cells (e) that express KR, or KQ but with the knockdown of *CXCR4*, as indicated by representative images of TRAP staining assay of the cocultures. (f) Knockdown of *IL11* attenuated KQ-expressing DU 145 cells to promote osteoclast differentiation, as indicated by TRAP staining of the cocultures of RAW264.7 cells and PCa cells. (g) Knockdown of *IL11* abolished the suppressive effects of *CXCR4* knockdown on osteoclast differentiation induced by KQ expressing DU 145 cells, as indicated by TRAP staining of the cocultures of RAW264.7 cells and PCa cells. A8 and A9 are two different shRNAs of *CXCR4*, and C3 and C6 are two different shRNAs of *IL11*. n=3 wells per group in **f** and **g**. (h) KQ expression in DU 145 or PC-3 cells increased IL-6 secretion in their co-cultures with MC3T3-E1 cells, as detected by ELISA. The ELISA were performed in triplicate. (i) CM from co-cultures of MC3T3-E1 cells with KQ cells further enhanced the osteoclast differentiation of RAW264.7 cells induced by KQ cells, as indicated by TRAP+ osteoclasts per well (left) and images of TRAP staining (right). n=3 wells per group. Black arrows indicate TRAP+ multinucleated osteoclasts. Scale bars, 50 µm. In panels, **b**, **c**, **f**-**i**, data are shown in mean ± S.E.M. *, $p < 0.05$; **, $p < 0.01$; ***, $p < 0.001$; NS, not significant (two-tailed Student's t-test). Source data are provided as a Source Data file.

Supplementary figure 9

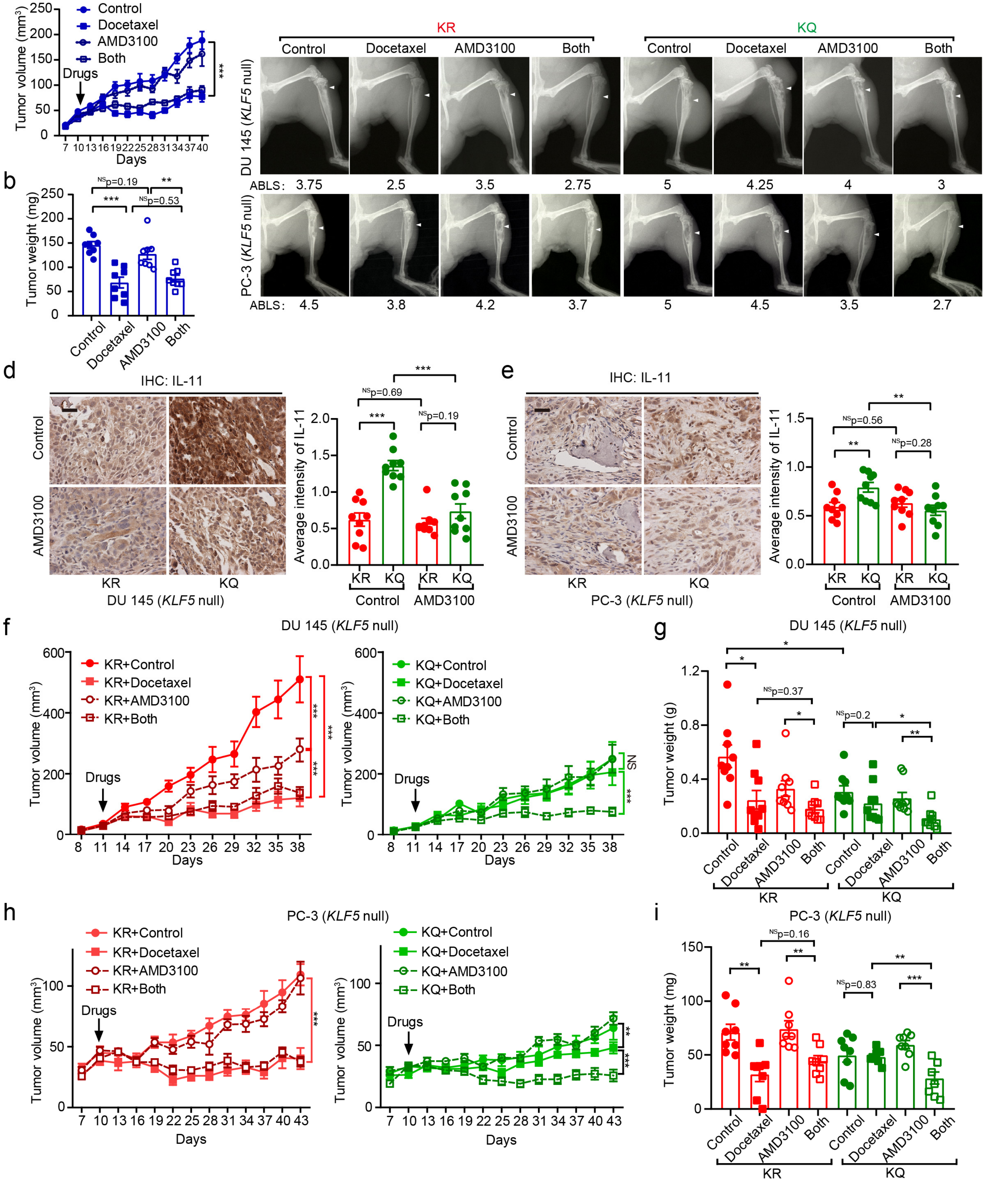

Supplementary figure 9. CXCR4 inhibitor AMD3100 sensitizes subcutaneous PCa tumors with Ac-KLF5 to docetaxel treatment. (a, b) Subcutaneous tumors of PC-3 parental cells were treated with docetaxel and/or AMD3100,

and tumor growth was indicated by tumor volume-based growth curves (a) and tumor weights at the endpoint (b). n=8 tumors per group. (c) X-ray radiographs of tibias at 38 days (DU 145) or 43 days (PC-3) after inoculation of cells expressing $KLF5^{KR}$ (KR) or $KLF5^{KQ}$ (KQ) into tibias. White arrows point to areas of bone lesions. Number under each panel are the **A**verage **B**one **L**esion **S**core (ABLS) based on the degree of osteolysis. (d, e) IHC staining of IL-11 in DU 145 (d) and PC-3 (e) tibial tumors expressing $KLF5^{KR}$ (KR) or $KLF5^{KQ}$ (KQ) with or without AMD3100 treatment. Scale bars, 50 µm. Nine images from three different tumors per group were used for statistical analysis. (f-i) Subcutaneous tumors of DU 145 (f, g) and PC-3 (h, i) cells expressing $KLF5^{KQ}$ (KQ) or $KLF5^{KR}$ (KR) were treated with docetaxel and/or AMD3100, and tumor growth was indicated by tumor volume-based growth curves (f, h) and tumor weights at the endpoint (g, i). n=9 tumors for DU 145, and n=8 tumors for PC-3. Treatments with docetaxel (10 mg/kg twice a week via i.p.) and/or AMD3100 (3.5 mg/kg/day via i.p.) started at day 11 after tumor inoculation. In panels **b**, **d**, **e**, **g** and **i**, data are shown in mean ± S.E.M. NS, not significant; *, $p < 0.05$; **, $p < 0.01$; ***, $p<0.001$ (two-tailed Student's t-test). In panels **a**, **f** and **h**, NS, not significant; **, $p < 0.01$; ***, $p<0.001$ (two-way ANOVA test). Source data are provided as a Source Data file.

Supplementary figure 10

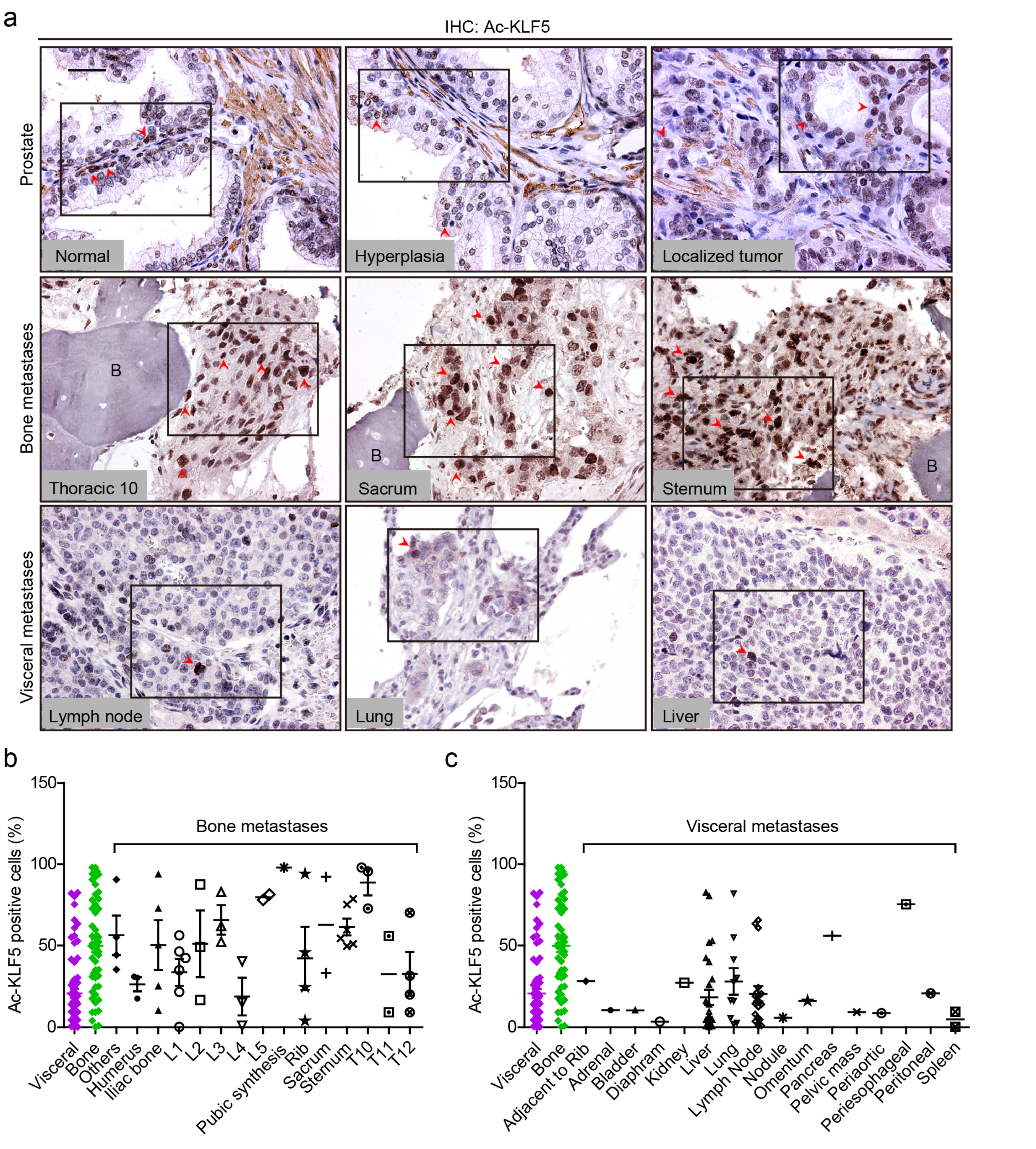

Supplementary figure 10. Expression of Ac-KLF5 in bone metastases and visceral metastases from PCa patients. (a) Representative images of IHC staining of Ac-KLF5 in benign tissues, hyperplasia and localized tumors of the prostate and metastases of PCa from both the visceral and bone tissues. Red arrows indicate positive Ac-KLF5 staining. Black rectangles indicate the regions which are zoomed in in **Figure 9a**. Scale bar, 50 µm. Representative images shown in panel **a** are statistically analyzed in **Figure 9b**. (b, c) Shown are percentages of Ac-KLF5-positive cells in different sites of

bone metastases (b) and visceral metastases (c) of PCa patients, as detected by IHC staining of Ac-KLF5 in tissue microarrays and quantitative analysis of Ac-KLF5-positive cells using the Fiji software. Seventy visceral metastases and 51 bone metastases from PCa patients are shown in purple and green, respectively, in panel **b** and **c** as references for expression quantification. Black dots indicate bone metastases of different sites in panel **b** and visceral metastases of different tissues/organs in panel **c**. n=4, 3, 5, 6, 3, 3, 3, 2, 1, 4, 2, 6, 3, 2, 4 different bone metastasis samples for Other bone metastases, Humerus, Iliac bone, L1, L2, L3, L4, L5, Pubic Synthesis, Rib, Sacrum, Sternum, T10, T11 and T12, respectively (**b**). n=1, 1, 1, 1, 1, 27, 10, 19, 1, 1, 1, 1, 1, 1, 1, 2 metastasis samples in different tissues including Adjacent to Rib, Adrenal, Bladder, Diaphram, Kidney, Liver, Lung, Lymph Node, Nodule, Omentum, Pancreas, Pelvic mass, Periaortic, Periesophageal, Peritoneal and Spleen, respectively (**c**). Data are shown in mean ± S.E.M.

II. Supplementary tables

Supplementary table 1. Primers used for realtime qPCR

Primer name	Primer sequences
Human-E-cadherin-for	5’-TGAAGGTGACAGAGCCTCTGGAT-3’
Human-E-cadherin-rev	5’-TGGGTGAATTCGGGCTTGTT-3’
Human-vimentin-for	5’-CAGGCGATATATTACCCAGGCAAG-3’
Human-vimentin-rev	5’-CTTGTAGGAGTGTCGGTTGTTAAG-3’
Human-N-cadherin-for	5’-GACAATGCCCCTCAAGTGTT-3’
Human-N-cadherin-rev	5’-CCATTAAGCCGAGTGATGGT-3’
Human-ZEB1-for	5’-TGCACTGAGTGTGGAAAAGC-3’
Human-ZEB1-rev	5’-TGGTGATGCTGAAAGAGACG-3’
Human-FN1-for	5’-CCATAAAGGGCAACCAAGAG-3’
Human-FN1-rev	5’-ACCTCGGTGTTGTAAGGTGG-3’
Human-GAPDH-for	5’-GGTGGTCTCCTCTGACTTCAACA-3’
Human-GAPDH-rev	5’-GTTGCTGTAGCCAAATTCGTTGT-3’
Mouse-KLF5-for	5’-AAGGAGTAACCCCGATTTGG-3’
Mouse-KLF5-rev	5’-CAGCCTTCCCAGGTACACTT-3’
Mouse-Trap-for	5’-CACTCCCACCCTGAGATTTGTG-3’
Mouse-Trap-rev	5’-ACGGTTCTGGCGATCTCTTTG-3’
Mouse-Nfatc1-for	5’-AAGTCTCACCACAGGGCTCACT-3’
Mouse-Nfatc1-rev	5’-CAAGTAACCGTGTAGCTGCACAAT-3’
Mouse-Itgb3-for	5’-CCTTTGCCCAGCCTTCCA-3’
Mouse-Itgb3-rev	5’-GTCCCCACAGTTACATTG-3’
Mouse-c-Myc-for	5’-TGAGCCCCTAGTGCTGCAT-3’
Mouse-c-Myc-rev	5’-TCCACAGACACCACATCAATTTC-3’
Mouse-Tm7sf4-for	5’-TGGGTGCTGTTTGCCGCTGT-3’
Mouse-Tm7sf4-rev	5’-TGGGTTCCTTGCTTCTCTCCACG-3’
Mouse-Ctsk-for	5’-AGAGAGCAGTGGCGCGGGTA-3’
Mouse-Ctsk-rev	5’-CCAGCTCTCTCCCCAGCTGTT-3’
Mouse-Mmp9-for	5’-GTTTTTGATGCTATTGCTGAGATCCA-3’
Mouse-Mmp9-rev	5’-CCCACATTTGACGTCCAGAGAAGAA-3’
Mouse-c-Src-for	5’-CTCCCGCACCCAGTTCAA-3’
Mouse-c-Src-rev	5’-GCCATCAGCATGTTTGGAGTAGT-3’
Mouse-Gapdh-for	5’-CCAGCCTCGTCCCGTAGACA-3’
Mouse-Gapdh-rev	5’-GCCGTTGAATTTGCCGTGAG-3’
Mouse-Il-6-for	5’-CAAGAGACTTCCATCCAGTTGCCT-3’
Mouse-Il-6-rev	5’-TTTCTCATTTCCACGATTTCCCAG-3’
Mouse-Osx-for	5’-CCCTTCTCAAGCACCAATGG-3’
Mouse-Osx-rev	5’-AGGGTGGGTAGTCATTTGCATAG-3’
Mouse-Runx2-for	5’-AAATGCCTCCGCTGTTATGAA-3’
Mouse-Runx2-rev	5’-GCTCCGGCCCACAAATCT-3’
Mouse-Bglap-for	5’-TTCTGCTCACTCTGCTGACC-3’
Mouse-Bglap-rev	5’-ACCTTATTGCCCTCCTGCTT-3’
Human-SHH-for	5’-CTCGCTGCTGGTATGCTCG-3’
Human-SHH-rev	5’-ATCGCTCGGAGTTTCTGGAGA-3’
Human-WNT5A-for	5’-GCCAGTATCAATTCCGACATCG-3’
Human-WNT5A-rev	5’-TCACCGCGTATGTGAAGGC-3’
Human-IL-11-for	5’-TGAAGACTCGGCTGTGACC-3’
Human-IL-11-rev	5’-CCTCACGGAAGGACTGTCTC-3’

Human-IL-6-for	5'-ACTCACCTCTTCAGAACGAATTG-3'
Human-IL-6-rev	5'-CCATCTTTGGAAGGTTCAGGTTG-3'
Human-CCL5-for	5'-CCAGCAGTCGTCTTTGTCAC-3'
Human-CCL5-rev	5'-CTCTGGGTTGGCACACACTT-3'
Human-ICAM1-for	5'-ATGCCCAGACATCTGTGTCC-3'
Human-ICAM1-rev	5'-GGGGTCTCTATGCCCAACAA-3'
Human-TGFB2-for	5'-CAGCACACTCGATATGGACCA-3'
Human-TGFB2-rev	5'-CCTCGGGCTCAGGATAGTCT-3'
Human-CSF1-for	5'-TGGCGAGCAGGAGTATCAC-3'
Human-CSF1-rev	5'-AGGTCTCCATCTGACTGTCAAT-3'
Human-TGFB1-for	5'-CAATTCCTGGCGATACCTCAG-3'
Human-TGFB1-rev	5'-GCACAACTCCGGTGACATCAA-3'
Human-MMP9-for	5'-ACGTGAACATCTTCGACGCCATC-3'
Human-MMP9-rev	5'-TCAGAGAATCGCCAGTACTTCCC-3'
Human-DLL4-for	5'-GTCTCCACGCCGGTATTGG-3'
Human-DLL4-rev	5'-CAGGTGAAATTGAAGGGCAGT-3'
Human-LIF-for	5'-TGTTTCCAGTGCAGAACCAA-3'
Human-LIF-rev	5'-GCATCTGAGGTTTCCTCCAA-3'
Human-WNT2B-for	5'-GGGGCACGAGTGATCTGTG-3'
Human-WNT2B-rev	5'-GCATGATGTCTGGGTAACGCT-3'
Human-PTHLH-for	5'-ACTCGCTCTGCCTGGTTAGA-3'
Human-PTHLH-rev	5'-GGAGGTGTCAGACAGGTGGT-3'
Human-GNRH1-for	5'-CAAAAACTCCTAGCTGGCCTT-3'
Human-GNRH1-rev	5'-CAGTTGACCAACCTCTTTGACT-3'
Human-IL-18-for	5'-ATCGCTTCCTCTCGCAACAA-3'
Human-IL-18-rev	5'-CTTCTACTGGTTCAGCAGCCATCT-3'

Supplementary table 2. Primers used for CXCR4 promoter cloning

Primer name	Primer sequences
pCXCR4 (-785)-for	GGGGTACCCCACGGTGTTGTGAGAATGA
pCXCR4 (+137)-rev	CCGCTCGAGCGGAAGCAGGTTGAAACTGGACT
pCXCR4 (-550)-for	GGGGTACCCCACCTGACCCTAGTGATGC
pCXCR4 (-312)-for	GGGGTACCCACTTTAGCAAGGATGGACGC
pCXCR4 (-177)-for	GGGGTACCCCGACCACCCGCAAACAGC
pCXCR4 (-104)-for	GGGGTACCCCTTCCTCGCGTCTGCCCCTC
pCXCR4 (-71)-for	GGGGTACCCCTTCTCCCTCCCCGCCC
pCXCR4 (-46)-for	GGGGTACCCCATGCGCCGCGCTCGGAGCGTG

Supplementary table 3. Primers used for ChIP-qPCR

Primer name	Primer sequences
pCXCR4 (-550)-for	CACCTGACCCTAGTGATGC
pCXCR4 (-379)-rev	GCTGGAGTAACCCAACCA
pCXCR4 (-312)-for	ACTTTAGCAAGGATGGACGC
pCXCR4 (-168)-rev	CGGGTGGTCGGTAGTGAG
