# Supplementary material for: Acetylation of KLF5 maintains EMT and tumorigenicity to cause chemoresistant bone metastasis in prostate cancer
Source: Nat Commun. 2021 Mar 17;12:1714. doi: 10.1038/s41467-021-21976-w (PMC7969754; doi:10.1038/s41467-021-21976-w)
Supplement: Supplementary file 3 — Description of Additional Supplementary Files [file 41467_2021_21976_MOESM3_ESM.docx]

**File Name:** Supplementary data 1

**Description:** FPKM of DU 145 cells with different forms of KLF5 in the RNA-Seq analysis, corresponding to Supplementary Figures 7a and 7b.

**File Name:** Supplementary data 2

**Description:** FPKM of PC-3 cells with different forms of KLF5 in the RNA-Seq analysis, corresponding to Supplementary Figures 7c and 7d.

**File Name:** Supplementary data 3

**Description:** Comparison of genes regulated by KLF5 in DU 145 and PC-3 cells, as identified by the RNA-Seq analysis, corresponding to Supplementary Figure 7e (black dots).

**File Name:** Supplementary data 4

**Description:** Genes regulated by KLF5 acetylation in both DU 145 and PC-3 cells, as identified by the RNA-Seq analysis, corresponding to Supplementary Figure 7f (black dots).

**File Name:** Supplementary data 5

**Description:** Promoter binding regions by different forms of KLF5 in DU 145 cells, as detected by the ChIP-Seq analysis, corresponding to Supplementary Figures7g and 7h.

**File Name:** Supplementary data 6

**Description:** Overlapping genes between RNA-Seq and ChIP-Seq analyses in the EV and KLF5 groups of DU 145 cells, corresponding to Supplementary Figure 7i.

**File Name:** Supplementary data 7

**Description:** Overlapping genes between RNA-Seq and ChIP-Seq analyses in the KR and KQ groups of DU 145 cells, corresponding to Figure 5a.

**File Name:** Supplementary data 8

**Description:** Nonoverlapping genes between RNA-Seq and ChIP-Seq analyses in the EV and KLF5 groups of DU 145 cells, corresponding to Supplementary Figure 5j.

**File Name:** Supplementary data 9

**Description:** Nonoverlapping genes between RNA-Seq and ChIP-Seq analyses in the KR and KQ group of DU 145 cells, corresponding to Supplementary Figure 7k.
