# Supplementary material for: Acetylation of KLF5 maintains EMT and tumorigenicity to cause chemoresistant bone metastasis in prostate cancer
Source: Nat Commun. 2021 Mar 17;12:1714. doi: 10.1038/s41467-021-21976-w (PMC7969754; doi:10.1038/s41467-021-21976-w)
Supplement: Supplementary file 13 — Reporting Summary [file 41467_2021_21976_MOESM13_ESM.pdf]

nature research

Corresponding author(s): Jin-Tang Dong

Last updated by author(s): 12/18/2020

Reporting Summary

Nature Research wishes to improve the reproducibility of the work that we publish. This form provides structure for consistency and transparency in reporting. For further information on Nature Research policies, see our Editorial Policies and the Editorial Policy Checklist.

Statistics

For all statistical analyses, confirm that the following items are present in the figure legend, table legend, main text, or Methods section.

n/a	Confirmed	
☐	☒	The exact sample size (*n*) for each experimental group/condition, given as a discrete number and unit of measurement
☐	☒	A statement on whether measurements were taken from distinct samples or whether the same sample was measured repeatedly
☐	☒	The statistical test(s) used AND whether they are one- or two-sided<br>*Only common tests should be described solely by name; describe more complex techniques in the Methods section.*
☒	☐	A description of all covariates tested
☐	☒	A description of any assumptions or corrections, such as tests of normality and adjustment for multiple comparisons
☐	☒	A full description of the statistical parameters including central tendency (e.g. means) or other basic estimates (e.g. regression coefficient) AND variation (e.g. standard deviation) or associated estimates of uncertainty (e.g. confidence intervals)
☐	☒	For null hypothesis testing, the test statistic (e.g. *F*, *t*, *r*) with confidence intervals, effect sizes, degrees of freedom and *P* value noted<br>*Give P values as exact values whenever suitable.*
☒	☐	For Bayesian analysis, information on the choice of priors and Markov chain Monte Carlo settings
☒	☐	For hierarchical and complex designs, identification of the appropriate level for tests and full reporting of outcomes
☐	☒	Estimates of effect sizes (e.g. Cohen's *d*, Pearson's *r*), indicating how they were calculated

Our web collection on statistics for biologists contains articles on many of the points above.

Software and code

Policy information about availability of computer code

Data collection	Fiji Software (version image J-win64) was used to count positive staining cells in IHC staining. Fiji is an upgrade version of Image J program, and available online at "https://imagej.net/Fiji". We used Plugins\Analyze\Cell counter for counting the cell numbers and Analyze\measure to quantify staining signal intensities. Sphere numbers were also counted similarly by Fiji software.
Data analysis	GraphPad Prism 8 software (v8.0.1), which is commercially available, was used for data analysis. Flow cytometry data was plotting using Flowjo software (v7.6.1). FASTQ files of sequencing were quality controlled and adapter trimmed using FASTQC (v0.11.5) prior to mapping them to the HG19 reference genome using bowtie (v2.2.6). Enriched regions were determined using MACS2 (v2.1.0.20151222). The union of all enriched regions was determined and coverage in these regions were determined using the 'summarizeOverlaps' function in R/ Bioconductor (v3.6.1) prior to differential analysis using edgeR (v3.26.5).

For manuscripts utilizing custom algorithms or software that are central to the research but not yet described in published literature, software must be made available to editors and reviewers. We strongly encourage code deposition in a community repository (e.g. GitHub). See the Nature Research guidelines for submitting code & software for further information.

Data

Policy information about availability of data

All manuscripts must include a data availability statement. This statement should provide the following information, where applicable:

- Accession codes, unique identifiers, or web links for publicly available datasets
- A list of figures that have associated raw data
- A description of any restrictions on data availability

The data generated or analyzed during the current study are available within the article, supplementary information, attached source data file, and from the corresponding author upon reasonable request. The source data underlying figures 1-9 and supplementary figures 1-10 are provided as a Source Data file. The RNA-

Seq and ChIP-Seq data that support the findings of this study have been deposited in Gene Expression Omnibus (GEO) with an accession code of GSE161951 (https://www.ncbi.nlm.nih.gov/geo/query/acc.cgi?acc=GSE161951), which is linked to two SubSeries, GSE161949 for ChIP-Seq data and GSE161950 for RNA-Seq data.

Field-specific reporting

Please select the one below that is the best fit for your research. If you are not sure, read the appropriate sections before making your selection.

☒ Life sciences ☐ Behavioural & social sciences ☐ Ecological, evolutionary & environmental sciences

For a reference copy of the document with all sections, see nature.com/documents/nr-reporting-summary-flat.pdf

Life sciences study design

All studies must disclose on these points even when the disclosure is negative.

Sample size	For animal experiments, sample size calculation was based on the multiple two-sample tests with alpha level being split by the number of tests in each experiment. Setting the statistical power at 80% and an overall alpha level of 0.05 and using a two-sided test. For in vitro experiments, no sample size calculation was performed and we generally included three samples per condition.
Data exclusions	Some samples in the tissue microarray (TMA) were torn and/or generated a dark nonspecific background, and had to be excluded from analyses.
Replication	Realtime qPCR were performed in duplicate or triplicate as indicated in the figure legends. Flow cytometry and promoter reporter assay were performed in triplicate. In vitro experiments using cell lines were repeated at least twice. Consistent results were achieved. IHC staining assays were not repeated with the same slides, instead we included different samples as we described in the figure legends. The IHC staining of different samples were statistically analyzed together. RNA-Seq and ChIP-Seq were not repeated, we validated them in the following in vitro assays.
Randomization	For animal experiments, we randomly grouped the tumor bearing animals before treatment. For the IHC staining images, pictures were taken from randomly selected fields. For the images of sphere and coculture assays, pictures were also taken randomly.
Blinding	Quantification of IHC and X-ray scoring data were done by a person who was blinded to genotypes and treatments of samples. Measurement of the tumor growth subcutaneously and in tibia was performed blindly by other colleagues or core facilities. Analyses of RNA-Seq and ChIP-Seq were performed by the researchers in other lab who were blinded to the genotypes. Analysis of in vitro coculture assays were also read by a colleague who was blinded to the treatment conditions.

Reporting for specific materials, systems and methods

We require information from authors about some types of materials, experimental systems and methods used in many studies. Here, indicate whether each material, system or method listed is relevant to your study. If you are not sure if a list item applies to your research, read the appropriate section before selecting a response.

Materials & experimental systems

n/a	Involved in the study	
☐	☒	Antibodies
☐	☒	Eukaryotic cell lines
☒	☐	Palaeontology and archaeology
☐	☒	Animals and other organisms
☐	☒	Human research participants
☒	☐	Clinical data
☒	☐	Dual use research of concern

Methods

n/a	Involved in the study	
☐	☒	ChIP-seq
☐	☒	Flow cytometry
☒	☐	MRI-based neuroimaging

Antibodies

Antibodies used	All of the antibodies used in this study are described in the Methods section, including commercially available antibodies for E-cadherin (Cell Signaling, #3195), vimentin (Cell signaling, #5741), N-cadherin (BD Biosciences, #610920), β-actin (Sigma, #A2066), Ki67 (Thermo Fisher, #RM9106s), c-caspase 3 (Cell Signaling, #9661s), PE-conjugated anti-CXCR4 antibody (Biolegend, #306506), and PE-conjugated anti-mouse IgG2α, κ Isotype (Biolegend, #400211). Antibodies against KLF5 and Ac-KLF5 for Western blotting and IHC staining are generated and validated in our previous studies (Chen, C. et al. Oncogene 24:3319-27, 2005; Guo, P. et al. J Biol Chem 284:6071-6078, 2009; Zhang, B. et al. Nature Communicaitons 11, 2020). Antibodies against KLF5 for ChIP-Seq is commercially available at R&D (#AF3758). The antibody against CXCR4 for IHC staining is from Abcam (#ab124824) and Millipore (#AB1846). EnVision Polymer-HRP secondary antibodies were from Dako (#K4003).
Validation	The antibodies for E-cadherin (Cell Signaling, #3195) and vimentin (Cell signaling, #5741) were validated by Cell signaling technology for Western blotting, IF and IHC in both mouse and human cells or tissues (https://www.cellsignal.com/products/primary-antibodies/

e-cadherin-24e10-rabbit-mab/3195)(https://www.cellsignal.com/products/primary-antibodies/vimentin-d21h3-xp-rabbit-mab/5741?site-search-type=Products&N=4294956287&Ntt=vimentin&fromPage=plp). The antibody for N-cadherin (BD Biosciences, #610920) was validated by BD Biosciences for Western blotting in both human and mouse (https://www.bdbiosciences.com/us/applications/research/stem-cell-research/cancer-research/human/purified-mouse-anti-n-cadherin-32n-cadherin/p/610920). β-actin (Sigma, #A2066) was validated by Sigma-Aldrich for Western blotting in both human and mouse (https://www.sigmaaldrich.com/catalog/product/sigma/a2066?lang=en&region=US). Ki67 (Thermo Fisher, #RM9106s) was validated by Fisher Scientific for IHC staining in human (https://www.fishersci.com/shop/products/anti-ki-67-clone-sp6-thermo-scientific-lab-vision-1ml-unlabeled-supernatant/rm9106s). c-caspase 3 (Cell Signaling, #9661s) was validated by Cell signaling technology for Western blotting, IF and IHC in both mouse and human (https://www.cellsignal.com/products/primary-antibodies/cleaved-caspase-3-asp175-antibody/9661?site-search-type=Products&N=4294956287&Ntt=%239661s&fromPage=plp&_requestid=3215918). PE-conjugated anti-CXCR4 antibody (Biolegend, #306506) was validated by Biolegend for flow cytometry in human (https://www.biolegend.com/en-us/products/pe-anti-human-cd184-cxcr4-antibody-542). The antibody against CXCR4 (#ab124824) for IHC staining is validated by Abcam for IHC staining in human (https://www.abcam.com/cxcr4-antibody-umb2-ab124824.html). The antibodies of KLF5 and Ac-KLF5 were validated in our lab as described in our previous publications (Chen, C. et al. Oncogene 24:3319-27, 2005; Guo, P. et al. J Biol Chem 284:6071-6078, 2009; Zhang, B. et al. Nature Communicaitons 11, 2020).

Eukaryotic cell lines

Policy information about cell lines

Cell line source(s)	Two prostate cancer cell lines, PC-3 and DU 145, were purchased from the American Type Culture Collection (ATCC, Manassas, VA). The C4-2B cell line was a gift of Dr. Leland Chung (Cedars-Sinai Medical Center, Los Angeles, CA), whose lab established this cell line in their previous study (Thalmann GN, et al. Canc Res 54(10):2577-2581, 1994). RAW264.7 cell line was also purchased from ATCC with a catalog number (ATCC® TIB-71).
Authentication	ATCC provided certificates and their authentication was confirmed by the Emory Integrated Genomics Core facility using the short tandem repeat (STR) analysis.
Mycoplasma contamination	Confirmed to be free of contamination.
Commonly misidentified lines (See ICLAC register)	None

Animals and other organisms

Policy information about studies involving animals; ARRIVE guidelines recommended for reporting animal research

Laboratory animals	BALB/c nude mice and NOD SCID mice (3–4 weeks old) were purchased from Charles River (San Diego, CA) and the Jackson Lab (Bar Harbor, ME) respectively. All of the mice used for analyses were male.
Wild animals	No wild animals were used in this study.
Field-collected samples	No Field collected samples were used in this study.
Ethics oversight	Use of mice at an Emory University Division of Animal Resources facility was approved by the Institutional Animal Care and Use Committee of Emory University.

Note that full information on the approval of the study protocol must also be provided in the manuscript.

Human research participants

Policy information about studies involving human research participants

Population characteristics	All the human tissue microarrays are from either US Biomax and Prostate Cancer Biorepository Network (PCBN). The patients characteristics information can be retrieved from the below link: https://www.biomax.us/tissue-arrays/Prostate/PR807c, https://www.biomax.us/PR808, https://www.biomax.us/tissue-arrays/Prostate/PR8011b and https://prostatebiorepository.org/specimens (upon request from PCBN by contacting Dr. Colm Morrissey at cmorriss@uw.edu)
Recruitment	Given all of the patients data are from publicly available tissue microarray, self-selection bias is excluded.
Ethics oversight	According to the information from US Biomax, each specimen collected from any clinic was consented to by both hospital and individual.(https://www.biomax.us/Support). The US Biomax follows standard medical care and protect the donors' privacy. The donor's identity is anonymity and each human tissue is identified by Code ID only. Collection protocol was completed under approval of Ethical Committee of each hospital. On the other side, high ethical and privacy standards to protect the participants from whom PCBN biospecimens are obtained. All network sites of the PCBN operate with the review and approval of their local Institutional Review Board (IRB). All rapid autopsy tissues were collected from patients who signed written informed consent under the aegis of the Prostate Cancer Donor Program at the University of Washington. The Institutional Review Board of the University of Washington approved this study (IRB#2341).

Note that full information on the approval of the study protocol must also be provided in the manuscript.

ChIP-seq

Data deposition

☒ Confirm that both raw and final processed data have been deposited in a public database such as GEO.

☒ Confirm that you have deposited or provided access to graph files (e.g. BED files) for the called peaks.

Data access links<br>*May remain private before publication.*	To review GEO accession GSE161951:<br>Go to https://www.ncbi.nlm.nih.gov/geo/query/acc.cgi?acc=GSE161951<br>Enter token ytkfeygydxkdtif into the box
Files in database submission	RAW data, BIGWIG files and a spreadsheet to summarize the peak comparison among samples.
Genome browser session<br>(e.g. UCSC)	IGV

Methodology

Replicates	One sample per genotype with input as controls
Sequencing depth	The DNA samples were sent to BGI for quality control, library construction (SE50), and sequencing with the BGISEQ-500 sequencer.
Antibodies	Antibodies against KLF5 for ChIP-Seq is commercially available at R&D (#AF3758).
Peak calling parameters	FASTQ files of sequencing were quality controlled and adapter trimmed using FASTQC (v0.11.5) prior to mapping them to the HG19 reference genome using bowtie (v2.2.6). Enriched regions were determined using MACS2 (v2.1.0.20151222) relative to input files with a q value (FDR adjusted p value) of 0.05. The union of all enriched regions was determined and coverage in these regions were determined using the 'summarizeOverlaps' function in R/Bioconductor (v3.6.1) prior to differential analysis using edgeR (v3.26.5) where regions with an FDR ≤0.05 were considered significant.
Data quality	Description as above in Peak calling parameters.
Software	Description as above in Peak calling parameters.

Flow Cytometry

Plots

Confirm that:

☒ The axis labels state the marker and fluorochrome used (e.g. CD4-FITC).

☒ The axis scales are clearly visible. Include numbers along axes only for bottom left plot of group (a 'group' is an analysis of identical markers).

☒ All plots are contour plots with outliers or pseudocolor plots.

☒ A numerical value for number of cells or percentage (with statistics) is provided.

Methodology

Sample preparation	Two dimensional cultured prostate cancer cells were dissociated using Trypsin-EDTA, collected and washed in sterile PBS. Single cell suspensions were stained with PE-conjugated anti-CXCR4 antibody (Biolegend, #306506) in cell staining buffer (Biolegend, #420201) in dark overnight at 4°C. PE-conjugated anti-mouse IgG2α, κ Isotype (Biolegend, #400211) was used as the control antibody.
Instrument	BD FACSCanto™ II Cell Analyzer
Software	Flowjo (v7.6.1)
Cell population abundance	Cells were not sorted in the current study
Gating strategy	Gating strategies were indicated in supplementary Fig. 8a.

☒ Tick this box to confirm that a figure exemplifying the gating strategy is provided in the Supplementary Information.

April 2020
